# Selective Arylation of 2-Bromo-4-chlorophenyl-2-bromobutanoate via a Pd-Catalyzed Suzuki Cross-Coupling Reaction and Its Electronic and Non-Linear Optical (NLO) Properties via DFT Studies

**DOI:** 10.3390/molecules25153521

**Published:** 2020-07-31

**Authors:** Usman Nazeer, Nasir Rasool, Aqsa Mujahid, Asim Mansha, Muhammad Zubair, Naveen Kosar, Tariq Mahmood, Ali Raza Shah, Syed Adnan Ali Shah, Zainul Amiruddin Zakaria, Muhammad Nadeem Akhtar

**Affiliations:** 1 Department of Chemistry, Government College University, Faisalabad 38000, Punjab, Pakistan; usmannazeer1187@gmail.com (U.N.); aqsamujahid99@yahoo.com (A.M.); mansha.asim@gmail.com (A.M.); zubairmkn@yahoo.com (M.Z.); razagcu756@gmail.com (A.R.S.); 2 College of Chemistry and Molecular Engineering, Zhengzhou University, Kexue road No. 100, Zhengzhou 450001, China; 3 Department of Chemistry, COMSATS University Islamabad, Abbottabad Campus, University Road, Tobe Camp, Abbottabad 22060, Pakistan; naveenflavia@gmail.com (N.K.); mahmood@cuiatd.edu.pk (T.M.); 4 Faculty of Pharmacy, Universiti Teknologi MARA Cawangan Selangor Kampus Puncak Alam, Bandar Puncak Alam 42300, Malaysia; syedadnan@uitm.edu.my; 5 Atta-ur-Rahman Institute for Natural Products Discovery (AuRIns), Universiti Teknologi MARA Cawangan Selangor Kampus Puncak Alam, Bandar Puncak Alam 42300, Malaysia; 6 Department of Biomedical Science, Faculty of Medicine and Health Sciences, Universiti Putra Malaysia, Serdang 43400, Malaysia; 7 Halal Institute Research Institute, Universiti Putra Malaysia, Serdang 43400, Malaysia; 8 Faculty of Industrial Sciences & Technology, Universiti Malaysia Pahang, Lebuhraya Tun Razak, Gambang Kuantan 26300, Malaysia; nadeemupm@gmail.com; 9 Bio-Aromatic Research Center of Excellence, Faculty of Industrial Sciences & Technology, Universiti Malaysia Pahang, Lebuhraya Tun Razak, Gambang Kuantan 26300, Malaysia

**Keywords:** suzuki cross coupling, frontier molecular orbital analysis, molecular electrostatic potential, non-linear optical properties

## Abstract

In the present study, 2-bromo-4-chlorophenyl-2-bromobutanoate (**3**) was synthesized via the reaction of 2-bromo-4-chlorophenol with 2-bromobutanoyl bromide in the presence of pyridine. A variety of 2-bromo-4-chlorophenyl-2-bromobutanoate derivatives (**5a**–**f**) were synthesized with moderate to good yields via a Pd-catalyzed Suzuki cross-coupling reaction. To find out the reactivity and electronic properties of the compounds, Frontier molecular orbital analysis, non-linear optical properties, and molecular electrostatic potential studies were performed.

## 1. Introduction

Carboxylic esters are an important class of organic compounds, having various biological activities such as antifungal [1], antioxidant [2], anti-inflammatory [3], antiproliferative [4], and anti-bacterial [5], and they have been widely used in the biofuel [6], plasticizer [7], food, and cosmetic industries [8]. In natural product chemistry, esters are well known common intermediates because of their accessibility for easy interconversion and their stability. Esters are generally synthesized by the condensation of carboxylic acids with alcohols. Moreover, activation of the carboxyl group followed by reaction with suitable alcohol is one of the most familiar methods for ester formation [9]. Similarly, biphenyl compounds are used as central building blocks for basic liquid crystal [10] and fluorescent layers in organic light-emitting diodes (OLEDs) [11] and are also important pharmaceutically as anti-hypertensive [12], anti-inflammatory [13], potential anti-cholinesterase [14], anti-diabetic [15], anti-tumor [16], anti-cancer [17], and anti-leukemia agents [18], and as potential therapeutics for cardiovascular disease [19] and osteoporosis [20].

For the synthesis of biphenyl compounds, the Suzuki–Miyaura reaction is one of the best methods. In organic synthesis, regioselective functionalization plays a progressively important role [21]. In such types of reactions, the reaction takes place at the carbon atom, which is more electron-deficient, while the other active site remains unattached. This concept has been practiced in region-selective Pd-catalyzed cross-coupling reactions based on different rates of oxidative additions of Pd (0) species to carbon halide bonds of different substrates. Therefore, we focused on the synthesis of 2-bromo-4-chlorophenyl-2-bromobutanoate followed by regioselective arylation of 2-bromo-4-chlorophenyl-2-bromobutanoate via a palladium-catalyzed Suzuki–Miyaura cross-coupling reaction to obtain the 2-bromo-4-chlorophenyl-2-bromobutanoate derivatives. Frontier molecular orbital (FMO), non-linear optic properties (NLO) and molecular electrostatic potential (MEP) studies were performed to find out the most reactive site of the compound.

## 2. Results and Discussion

### 2.1. Chemistry

**Scheme 1:**
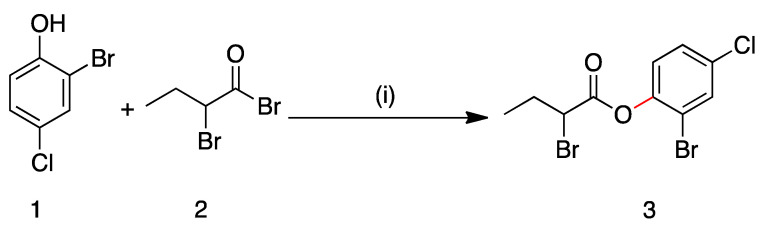
Synthesis of 2-bromo-4-chlorophenyl-2-bromobutyrate (3). Conditions: (i) **1** (1 g, 4.820 mmol), **2** (0.64 mL, 5.3 mmol), pyridine (0.7 mL, 9.63 mmol), MeCN (30 mL), 0 °C, 1 h.

In the present study, 2-bromo-4-chlorophenyl-2-bromobutyrate (**3**) was synthesized by the reaction of 2-bromo-4-chlorophenol (**1**) with 2-bromobutryl bromide (**2**) in the presence of pyridine and acetonitrile, and good yield of 2-bromo-4-chlorophenyl-2-bromobutyrate (**3**) was observed (Scheme 1).

**Scheme 2:**
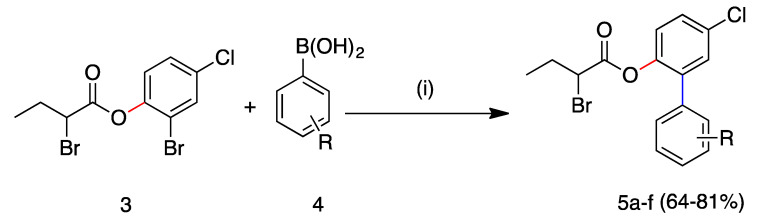
Synthesis of 2-aryl-4-chlorophenyl-2-bromobutyrates (5a–f). Conditions: (i) **3** (200 mg, 0.062 mmol, 1 eq), **4** (0.683 mmol, 1.1 eq), Pd (PPh_3_)_4_ (35 mg, 5 mol %), K_3_PO_4_ (263 mg, 1.24 mmol, 2.2 eq), 1, 4-dioxane (8 mL), water (4 mL), 90 °C, 17 h under argon.

In addition, the Suzuki–Miyaura cross-coupling reaction of 2-bromo-4-chlorophenyl-2-bromo butyrate (**3**) with different phenyl boronic acids was accomplished, which ultimately led to the formation of the corresponding 2-bromo-4-chlorophenyl-2-bromobutyrates (**5a**–**f**) in moderate to good yields (64–81%) (Scheme 2, Table 1). When 1.1 eq of phenyl boronic acid was used, it was noted that the bromo group of the phenyl group was replaced, while in the presence of Pd (PPh_3_)_4_, the other bromo group present at the alpha position could not be replaced. The chloro group at the phenyl ring was not replaced due to the C-Cl bond strength that obstructs the reactivity of aryl chlorides, thus, they are resistant to the oxidative addition to Pd(0) because activation of aryl chlorides is much more difficult than that of aryl bromides. The oxidative addition step is a selectivity-determining step in Suzuki cross-coupling, and it is broadly controlled by the bond dissociation energies of the carbon–halogen bond that predictably differs as a function of the aryl halides: Ar-I > Ar-Br > Ar-Cl > Ar-F [22]. It was noted that phenyl boronic acids having electron-donating substituents gave high yields as compared to electron-withdrawing substituted phenyl boronic acids.

### 2.2. Frontier Molecular Orbital Analysis

Frontier molecular orbital (FMO) analysis is well known to explain the reactivity and electronic properties of the molecules [23]. The energies of HOMOs (E_HOMOs_) and LUMOs (E_LUMOs_) provide information about the electronic transition and the HOMOs–LUMOs gap (G_H-L_) explains the reactivity and kinetic stability of the compounds. If the G_H-L_ is low, the compound will be less stable and more reactive, but if the G_H-L_ is large, the compound will be highly stable and less reactive [24]. The energies of HOMOs (E_HOMOs_), energies of LOMOs (E_LUMOs_), and the highest occupied molecular orbital to lowest unoccupied molecular orbital gap (G_H-L_) are summarized in Table 2.

Among all compounds, compound **5c** has the lowest G_H-L_ (4.27 eV) where the E_HOMOs_ and E_LUMOs_ were −6.06 and −1.79 eV, respectively. Based on the lower G_H-L_, **5c** is considered as kinetically less stable and moderately reactive. Compound **5f** has G_H-L_ of 5.19 eV, which showed it is the most stable and least reactive of the whole series (**5a**–**f**). The G_H-L_ of the other compounds were between 4.57 and 5.14 eV, which represent their moderate reactivity and sufficient stability. Although the G_H-L_ of **5c** is low, compared to other compounds, it is large.

**Figure 1:**
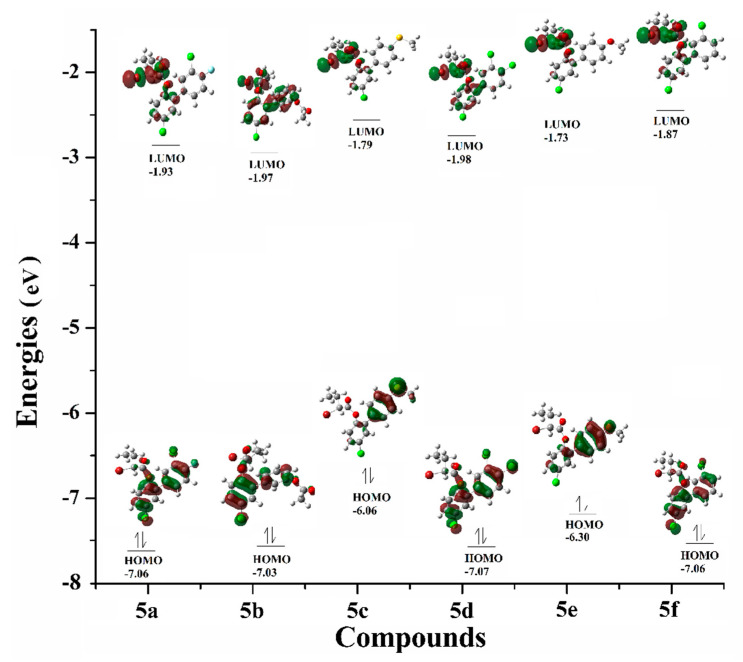
FMOs plot of all compounds (**5a**–**f**) based on the relative energies; the energies are in eV.

The iso-density distributions in the FMOs of all compounds were also shown in Figure 1. In all the compounds (except compounds **5c** and **5e**), the iso-density of HOMOs was distributed on the phenyl rings and halogen (chlorine and fluorine) groups that were present on the phenyl rings. However, this iso-density of HOMOs is shifted from the chlorine-containing phenyl ring towards the MeS- and MeO-groups containing phenyl rings in compounds **5c** and **5e**, respectively. This specific distribution explains the lower iso-density of **5c** and **5e** compared to that of the other compounds. The shift of iso-density is more pronounced for **5c** in comparison to **5e**. The iso-density of LUMOs is mostly distributed on the bromo-ester group attached to the phenyl ring in all compounds (**5a**–**f**).

### 2.3. Reactivity Descriptor Parameters

For further elucidation of the reactivity of these compounds (**5a**–**f**), some other reactivity descriptor parameters were also studied. The results of the analyses are given in Table 3. These parameters include ionization potential (*I*), electrophilicity index (Ꙍ), nucleophilicity (N), electron affinity (*E_A_*), electronic chemical potential (μ), and chemical hardness (Ƞ). According to Koopman’s theorem, the negative of HOMOs and LUMOs values correspond to the ionization potential (*I*) and electron affinity (*E_A_*), respectively [25].

#### 2.3.1. Ionization Potential, Electron Affinity, and Chemical Hardness (Ƞ)

The Chemical hardness is mathematically expressed as:

Chemical hardness (Ƞ) = (E_HOMO_ − E_LUMO_)/2 (1)

The values obtained for all compounds from different reactivity descriptor are comparable and support each other, which strengthens our findings. The ionization potential of all compounds was in the range of 6.06 eV to 7.06 eV. The most reactive and unstable compound in this series was **5c**, and its reactivity was also supported from its lower ionization potential (*I* = 6.06 eV), electron affinity (*E_A_* = 1.79 eV), and chemical hardness (Ƞ = 2.13 eV) values. The compound **5f** was the most stable compound, which is also confirmed from its high *I* (7.06 eV) and Ƞ (2.60 eV) values. The chemical hardness of the other compounds was between 2.13 and 2.57 eV.

#### 2.3.2. Electronic Chemical Potential (μ)

The electronic chemical potential gives us an idea about the charge transfer inside compounds at their ground state, and it is mathematically expressed as:

Electronic chemical potential (μ) = (E_HOMO_ + E_LUMO_)/2 (2)

The electronic chemical potential (μ) of all compounds (**5a**–**f**) was in the range of −3.92 eV to −4.52 eV. The highest μ (−4.52 eV) value was observed for **5d** and the lowest μ value (−3.92 eV) was calculated for **5c**.

#### 2.3.3. Electrophilicity Index (Ꙍ)

Parr et al. provided the concept of the electrophilicity index (Ꙍ) [26], which is mathematically expressed as:

Electrophilicity index (Ꙍ) = μ2/2Ƞ(3)

According to this concept, the Ꙍ is the stabilization of a compound based on energy when an additional charge is transferred from the surroundings or outside environment. The Ꙍ ranged from 3.52 eV to 4.02 eV for compounds **5a**–**f**. The compound **5e** had the lowest Ꙍ value (3.52 eV) in the series of compounds, which indicates that this compound is less stable for an incoming charge. The reason is the electronic acceptor and donor groups present in the compound are connected through extended conjugation. The incoming charge causes further delocalization of electronic density in the compound and enhances its reactivity.

#### 2.3.4. Nucleophilicity (N)

Nucleophilicity is derived from HOMOs and LUMOs values by using the following mathematical equation:

Nucleophilicity (N) = E_HOMO_ − E_LUMO_ (4)

Nucleophilicity is an important parameter for understanding the reactivity of compounds. All the compounds had nucleophilicity values in the range of 4.27 eV to 5.19 eV. The compound **5c** had the lowest *N* value (4.27 eV), which represents the soft nucleophilic nature of this compound. Whereas compound 3′5-Dichloro-[1,1′-biphenyl]-2-yl 2-bromobutanoate (**5f**) has the highest *N* value (5.19 eV), which shows the strong nucleophilic nature of this compound.

### 2.4. Molecular Electrostatic Potential (MEP)

**Figure 2:**
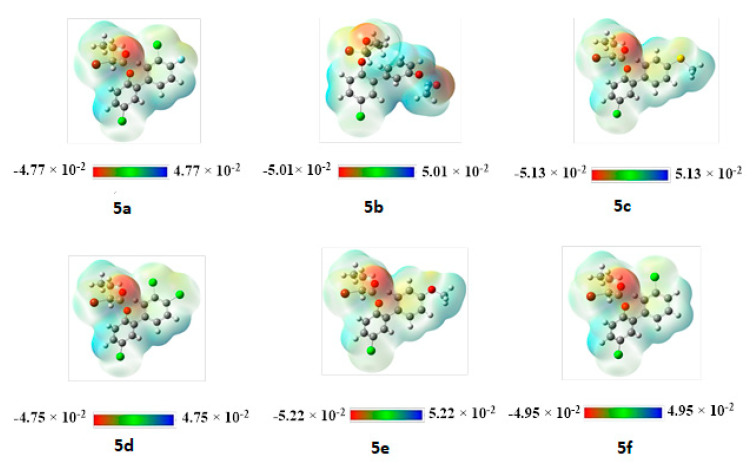
Molecular electrostatic potential surfaces of compounds (**5a**–**f**), the electronic density in red represents nucleophilic sites and in blue represents electrophilic site.

Molecular electrostatic potential (MEP) is used to evaluate and predict the reactive sites of a molecule [27]. MEP is generated on the surfaces of all optimized compounds to explain the nucleophilic and electrophilic sites. The potentials on the surfaces of complexes are characterized by various colors and are described in increasing order of red < orange < yellow < green < blue. The green colors show the neutral region while red and blue colors are used for the nucleophilic and electrophilic sites of the complex, respectively. The molecular electrostatic surfaces of all compounds are given in Figure 2 and the values are given in Table 4.

Figure 2 indicates that the ester group attached to the phenyl ring is an active site for an incoming electrophile. Whereas, hydrogen attached to the phenyl rings have blue electronic densities that represent the strong nucleophilic site. Yellow densities reside on the S atom of the MeS, O of the MeO, Cl, and F atoms, illustrating the moderately electrophilic sites of these functional groups. The highest electronic density distribution of −4.75 × 10^−2^ to 4.75 × 10^−2^ was observed for compound **5d**. In contrast, the lowest electronic density distribution of −5.22 × 10^−2^ to 5.22 × 10^−2^ was observed for compound **5e**.

### 2.5. Non-linear Optical (NLO) Properties

For the last decade, scientific society has been pursuing the pathways to synthesized non-linear optical (NLO) materials with large second-order nonlinear optical properties. Non-linear optical materials have various applications in laser devices, optical communication, optical data storage, optical limiting, optical computing, and medical imaging [28,29]. The NLO response of these materials is measured by the first hyperpolarizability (β_o_). In organic molecules, the movement of electrons from donor to acceptor group is responsible for the enhancement of β_o_, which ultimately results in a strong NLO response.

In the synthesized compounds (**5a**–**f**), the *para* chloro-phenyl ring (*p*-CPR) acts as electron-withdrawing group and influences different substitutions on the NLO response were studied at the ortho position of phenyl ring. The ester group is not involved due to the resonance between the two oxygen atoms and the electrons are unable to delocalize inside the phenyl ring. When the electron donating groups are attached to the *p*-CPR, the electron push full mechanism is the operative one and a large β_o_ value is obtained. However, if the electron-withdrawing groups are attached to the *p*-CPR, then the electron push full mechanism is not operating and the small β_o_ value is obtained in that case. The compound **5c** has a strong electron donating group (MeS-) at the para position of the phenyl ring and we noticed a large β_o_ value (1373.76 au) for it according to the push full mechanism. The lower β_o_ value (218.51 au) was observed for compound **5f** where an electron-withdrawing chloro group (Cl^−^) is present on the para position of the phenyl ring. On both sides of the phenyl rings, similar chloro-groups are attached which make these rings strongly electron-withdrawing, and the net effect is zero. The moderate electron donating methoxy group at the para position of the phenyl ring (in **5e**) had a reasonable NLO response compared to compound **5b** where the acetate group has an internal resonance, which has a negative impact on the NLO response. The β_o_ values of **5e** and **5b** compounds were 954.47 au and 284.34 au, respectively. Compounds **5a** and **5d** had almost comparable β_o_ values (in the range of 510 au −550 au). The reason may be that the presence of halogen groups on the *meta* and *para* positions of the ring nullified the effects of each other and the phenyl ring acted as moderately electron-donating. Based on these results, we suggest that compound **5c** shows a better NLO response and can be used as an active NLO material in the future.

The hyperpolarizability of these compounds (**5a**–**f**) was also supported by the polarizability values (Table 5). Polarizability is defined as the electronic density distribution in a system. In compounds where electron-accepting and electron-donating groups are present at the opposite termini of the phenyl rings, they must have positive and negative centers and their α_o_ is high. In contrast, in those compounds where both termini have similar functional groups, there is an equal distribution of electronic density and α_o_ is low. For example, compound **5c** had a high α_o_ value (264 au) whereas **5f** had a low α_o_ value (235 au). The rest of the compounds had moderate values of α_o_ in the range of 236 au to 252 au.

#### Non-linear Refractive Index (n_2_)

The quadratic non-linear refractive index (*n*_2_) was calculated by using degenerated four waves mixing gamma values (*γ*^DFWM^ (ω) = (−ω; ω, −ω, ω) as proposed by Tarazker et al. [30] and it is mathematically expressed as follows:

*γ*^DFWM^ (−ω; ω, −ω, ω) = (1/3) *γ* (−2ω; ω, ω, 0) + (1/3) *γ* (−ω; ω, 0, 0) + (1/3) *γ* (0; 0, 0, 0) (5)

where *γ* (0; 0, 0, 0), *γ* (−ω; ω, 0, 0), and *γ* (−2ω; ω, ω, 0) represent static, dc-Kerr, and EFSHG second hyperpolarizability coefficients, respectively. The *γ*^DFWM^ is further used to measure the quadratic non-linear refractive index (*n*_2_) using Equation 6.

*n*_2_ (cm^2^/W) = 8.28 × 10^−23^*γ*^DFWM^ (au) (6)

**Figure 3:**
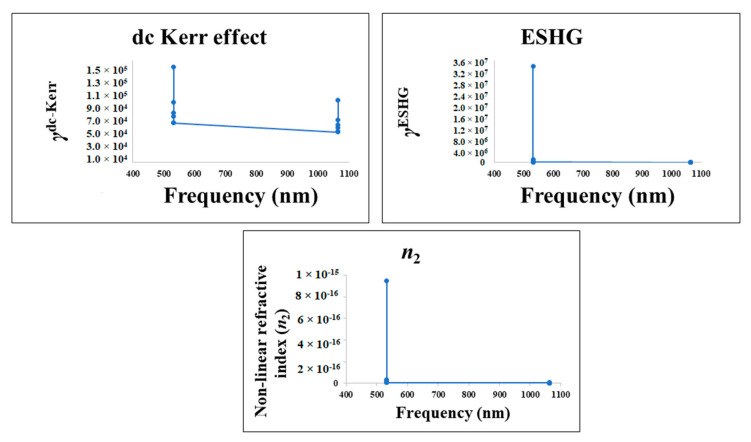
Graphical representations of dc-Kerr, ESHG coefficients, and refractive index for compounds (**5a**–**f**) at different wavelengths (532 and 1064 nm).

The *n*_2_ values of all compounds (**5a**–**f**) at various frequencies (0, 532 and 1064 nm) are given in Table 6 and graphically represented in Figure 2. In the spectral curves of *n*_2_, the ESHG effect and dc-Kerr effect are similar and demonstrate the dominant contribution of both these effects in enhancing *n*_2_ (Figure 3). However, the effect of the ESHG effect is more pronounced as compared to that of the dc-Kerr effect. The *n*_2_ values for compounds (**5a**–**f**) were in the range 8.01 × 10^−18^ au to 9.46 × 10^−16^ au at ω = 532nm. These compounds have *n*_2_ values in the range of 4.43 × 10^−18^ au to 8.41 × 10^−18^ au at ω = 1064 nm. Among all compounds, the distinct effect was noticed at a low wavelength (532 nm) where the highest value (9.46 × 10^−16^ au) was noticed for compound **5c**. These *n*_2_ values proved the influence of various wavelengths on the amplitude of hyperpolarizability and gamma values of these compounds. Besides the various wavelengths, the structure of the compounds also plays a substantial role in the fluctuation of the NLO response. The structure of compound **5c** has electron donating and electron withdrawing groups on both terminals of the main skeleton and an excellent push–pull mechanism occurs due to extended conjugation, and its NLO response is outstanding. This result is also supported by the nonlinear refractive index results.

## 3. Materials and Methods

### 3.1. Procedure for Synthesis of 2-Bromo-4-chlorophenyl-2-bromobutyrate (3)

First, 2-Bromo-4-chlorophenol (1 g, 4.820 mmol), MeCN (30 mL), pyridine (0.775 mL, 9.63 mmol), and 2-bromobutryl bromide (0.64 mL, 5.3 mmol) were added to a 250 mL round bottom flask with a magnetic stirrer. The flask’s mouth was closed with a Teflon septum. The flask was put on the magnetic hot plate along with an isotherm to maintain the temperature of the reaction mixture at 0 °C. The progress of the reaction was estimated by thin layer chromatography analysis. After 1 h, the reaction was completed. The separation and purification of the desired product was carried out using the column chromatography technique. The NMR spectroscopic technique was used to find out the structure of the desired compound [31].

*2-Bromo-4-chlorophenyl 2-bromobutanoate (**3**),* yellow liquid, ^1^H-NMR: (600 MHz, CDCl_3_) δ 7.9 (d, *J* = 1.5 Hz, 1H), 7.50 (dd, *J*_1_ = 7.5 Hz, *J*_2_ = 1.5 Hz, 1H), 7.29 (d, *J* = 7.5 Hz, 1H), 4.29 (t, *J* = 7.1 Hz, 1H), 2.05–2,10 (m, 2H, Ar), 1.1 (t, *J* = 8.0 Hz, 3H). ^13^C-NMR (150 MHz, CDCl_3_) δ 10.1 (CH_3_), 24.1 (CH_2_), 42.7 (CH), 115.2 (C), 117.6 (CH), 121.3 (CH), 129.4 (CH), 139.5 (C), 145.5 (C), 170.2 (C). HR-ESI-MS: *m/z* Calcd for C_10_H_9_Br_2_ClO, [M]^+^ 353.8909; found 353.8945.

### 3.2. General Procedure for Synthesis of (5**a**–**f**)

A reflux condenser flushed with argon was attached to a 100 mL schlenk flask with a magnetic stirrer. Then, 2-bromo-4-chlorophenyl-2-bromobutyrate (1 equiv.) and tetrakis(triphenyl phosphine)palladium(0) (5 equiv.) were added to the schlenk flask and dissolved into 8 mL of 1,4-dioxane and the flask’s mouth was closed with a Teflon septum. The reaction mixture was stirred for about 25–30 min under an inert argon atmosphere. Then, K_3_PO_4_ (2 equiv.) and aryl boronic acids and 2 mL distilled water were added and the reflux reaction continued under an inert argon atmosphere for 18–25 h at 80–90 °C. During the reaction, the estimation of the reaction was determined by a thin layer chromatography technique using TLC cards. When the reaction was completed, the reaction mixture was filtered using Whatman filter paper and the solvent was evaporated by a rotary evaporator. The separation and purification of the impure products were carried out using the column chromatography technique. ^1^H-NMR was used to find out the structure of the newly synthesized compounds [32].

*3′5-Dichloro-4′-fluoro-[1,1′-biphenyl]-2-yl 2-bromobutanoate (**5a**),* white crystals, m.p. 247–248 °C. ^1^H-NMR (600 MHz, CDCl_3_) 8.04 (d, *J* = 1.8 Hz, 1H), 8.00 (d, *J* = 1.5 Hz 1H), 7.55 (dd, *J*_1_ = 1.6 Hz, *J*_2_ = 7.7 Hz, 1H), 7.41 (dd, *J*_1_ = 7.7 Hz, *J*_2_ = 8.0 Hz 1H), 7.38 (d, *J* = 8.1 Hz, 1H), 7.35 (m, 1H), 4.2 (t, *J* = 7.0 Hz, 1H), 2.11–2.17 (m, 2H, Ar), 1.11 (t, *J* = 7.5 Hz, 3H). ^13^C-NMR (150 MHz, CDCl_3_) δ 10.2 (CH_3_), 25.4 (CH_2_), 42.7 (CH), 117.6 (CH), 122.4 (C), 125.6 (CH), 130.5 (CH), 131.6 (CH), 132.5 (CH), 133.2 (CH), 132.9 (C), 133.4 (C), 133.9 (C), 148.1 (C), 162.3 (C), 170.1 (C). HR-ESI-MS: *m/z* Calcd for C_16_H_12_BrCl_2_FO_2_, [M]^+^ 406.0722; found 406.0742.

*3′-Acetoxy-5-chloro-[1,1′-biphenyl]-2-yl 2-bromobutanoate (**5b**),* yellow crystals, m.p: 268–269 °C. ^1^H-NMR (600 MHz, CDCl_3_) δ 8.00 (d, *J* = 2.0 Hz, 1H), 7.50 (dd, *J*_1_ = 7.9 Hz, *J*_2_ = 1.8 Hz, 1H), 7.46 (d, *J* = 1.5 Hz, 1H), 7.42–7.34 (m, 2H), 7.35 (dd, *J*_1_ = 7.7 Hz, *J*_2_ = 1.5 Hz, 1H), 7.28 (d, *J* = 7.5 Hz, 1H), 4.20 (t, *J* = 7.0 Hz, 1H), 2.35 (s, 3H), 2.10–2.15 (m, 2H, Ar), 1.10 (t, *J* = 7.9 Hz, 3H). ^13^C-NMR (150 MHz, CDCl_3_) δ 10.1 (CH_3_), 19.4 (CH_3_), 25.6 (CH_2_), 45.4 (CH), 120.4 (CH), 121.1 (CH), 122.3 (CH), 128.7 (CH), 129.1 (CH), 131.2 (C), 132.7 (CH), 134.8 (CH), 136.3 (C), 137.5 (C), 145.1 (C), 148.1 (C), 170.1 (C), 171.2 (C). HR-ESI-MS: *m/z* Calcd for C_18_H_16_BrClO_4_, [M]^+^ 411.6808; found 411.6892.

*5-Chloro-4′-(methylthio)-[1,1′-biphenyl]-2-yl 2-bromobutanoate (**5c**),* brown crystals, m.p: 250–251 °C. ^1^H-NMR (600 MHz, CDCl_3_) δ 8.00 (d, *J* = 1.9 Hz, 1H), 7.65 (d, *J* = 8.2 Hz, 2H), 7.45 (d, *J* = 7.9 Hz, 2H), 7.35 (dd, *J*_1_= 7.5 Hz, *J*_2_ = 2.0 Hz, 1H), 7.25 (d, *J* = 8.4 Hz, 1H), 4.19 (t, *J* = 7.0 Hz, 1H), 2.53 (s, 3H), 2.05–2.11 (m, 2H, Ar), 1.10 (t, *J* = 7.0 Hz, 3H). ^13^C-NMR (150 MHz, CDCl_3_) δ 10.1 (CH_3_), 19.4 (CH_3)_, 25.6 (CH_2_), 45.4 (CH), 121.1 (CH), 124.3 (CH), 125.4 (2CH), 128.1 (C), 130.2 (CH), 131.1 (C), 132.7 (C), 134.8 (CH), 135.4 (CH), 136.1 (C), 146.1 (C), 170.1 (C). HR-ESI-MS: *m/z* Calcd for C_17_H_16_BrClO_2_S, [M]^+^ 399.7145; found 399.7119.

*3′,4′,5-Trichloro-[1,1′-biphenyl]-2-yl 2-bromobutanoate* (**5d**), white crystals, m.p: 276–277 °C. ^1^H-NMR (600 MHz, CDCl_3_) δ 7.96 (d, *J* = 2.3 Hz, 1H), 7.83 (d, *J* = 1.9 Hz, 1H), 7.51–7.43 (m, 2H), 7.40 (dd, *J*_1_ = 8.2 Hz, *J*_2_ = 2.0 Hz, 1H), 7.33 (d, *J* = 1.5 Hz, 1H), 4.20 (t, *J* = 7.0 Hz, 1H), 2.15–2.20 (m, 2H, Ar), 1.10 (t, *J* = 8.0 Hz, 3H).^13^C-NMR (150 MHz, CDCl_3_) δ 10.1 (CH_3_), 25.6 (CH_2_), 45.4 (CH), 121.1(CH), 126.5 (CH), 128.1 (CH), 130.2 (CH), 131.2 (CH), 132.7 (C), 135.8 (CH), 136.2 (C), 137.2 (C), 138.5 (C), 141.5 (C), 148.1 (C), 170.1 (C). HR-ESI-MS: *m/z* Calcd for C_16_H_12_BrCl_3_O_2,_ [M]^+^ 422.5233; found 422.5289.

*5-Chloro-4′-methoxy-[1,1′-biphenyl]-2-yl 2-bromobutanoate* (**5e**), light brown crystal, m.p: 237–238 °C. ^1^H-NMR (600 MHz, CDCl_3_) δ 7.98 (d, *J* = 2.0 Hz, 1H), 7.65 (d, *J* = 2.5 Hz, 2H) 7.49–7.35 (m, 2H), 7.10 (dd, *J*_1_ = 8.2 Hz, *J*_2_ = 1.5 Hz, 2H), 4.22 (t, *J* = 6.9 Hz, 1H), 3.93 (s, 3H), 2.08–2.15 (m, 2H, Ar), 1.10 (t, *J* = 8.0 Hz, 3H). ^13^C-NMR (150 MHz, CDCl_3_) δ 10.1 (CH_3_), 25.6 (CH_2_), 45.4 (CH), 60.2 (CH_3_), 116.1 (CH), 116.5 (CH), 124.5 (CH), 125.3 (C), 128.4 (CH), 130.3 (CH), 131.4 (C), 132.2 (CH), 13.3 (CH), 132.7 (C), 135.8 (C), 151.2 (C), 170.1 (C). HR-ESI-MS: *m/z* Calcd for C_17_H_16_BrClO_3_, [M]^+^ 383.4515; found 383.4565.

*3′,5-Dichloro-[1,1′-biphenyl]-2-yl 2-bromobutanoate* (**5f**), white crystal, m.p: 232–233 °C. ^1^H-NMR (600 MHz, CDCl_3_) δ 8.01 (d, *J* = 2.5 Hz, 2H), 7.52 (dd, *J*_1_ = 7.5 Hz, *J*_2_ = 1.9 Hz, 1H), 7.55 (t, *J* = 2.0 Hz, 1H), 7.42–7.41 (m, 2H), 7.34 (d, *J* = 7.0 Hz, 1H), 4.22 (t, *J* = 7.0 Hz, 1H), 2.08–2.15 (m, 2H, Ar), 1.10 (t, *J* = 8.0 Hz 3H). ^13^C-NMR (150 MHz, CDCl_3_) δ 10.1 (CH_3_), 25.6 (CH_2_), 45.4 (CH), 121.1 (2CH), 122.4 (CH), 121.9 (CH), 125.2 (CH), 128.7 (2CH), 130.2 (C), 132.7 (C), 133.2 (C), 134.7 (C), 151.3 (C), 171.2 (C). HR-ESI-MS: *m/z* Calcd for C_16_H_13_BrCl_2_O_2_, [M]^+^ 388.0861; found 388.0839.

### 3.3. Computational Details

All calculations were performed in Gaussian 09 software [33]. The optimized structures were drawn using Gauss View 5.0 software [34]. The structures of synthesized compounds (**5a**–**f**) were optimized with the hybrid functional (B3LYP) of the density functional theory (DFT). B3LYP is a widely used functional for the optimization of organic molecules because the minimization of errors in the geometrical parameters is lower for B3LYP compared to that of other density functionals. The split valence basis set developed by Pople’6-311G (d,p) was implemented for molecular orbital descriptions. For further confirmation of these minimum energy structures obtained from optimization (Figure 1), frequency analysis and frontier molecular orbital analysis were done at the same level of theory. During frequency simulations, the harmonic force constant was calculated, which validated the absence of imaginary frequency and justified these as true minima structures. FMOs, reactivity descriptor parameters (ionization potential, electron affinity, chemical hardness, electrophilicity index, electronic chemical potential, and nucleophilicity), and molecular electrostatic potential (MEP) were calculated with the B3LYP/6-311G (d,p) method. NLO properties including polarizabilities, first hyperpolarizability, and gamma values were calculated with CAM-B3LYP and LC-BLYP with 6-311+G (d,p) levels. The best NLO results were obtained by the CAM-B3LYP/6-31+G (d,p) level, so we discussed our results obtained on this level of theory. The nonlinear refractive index was also calculated a with the CAM-B3LYP/6-31+G (d,p) method.

## 4. Conclusions

In conclusion, a series of 2-bromo-4-chlorophenyl-2-bromobutyrate derivatives (**5a**–**f**) were synthesized and frontier molecular orbitals (FMOs) and molecular electrostatic potential (MEP) analysis were done. FMOs analysis revealed that among all the derivatives, **5c** is most reactive, having a HOMO–LUMI band gap of 4.27 eV, whereas the HOMO–LUMO band-gap for **5f** was 5.19 eV, and was the most stable. The chemical hardness of **5f** displayed the highest value (2.60 eV), whereas **5c** has the lowest value and was chemically soft and reactive. Among all the newly synthesized compounds, **5d** displayed the highest chemical potential value. The molecular electrostatic potential (MEP) investigation gave us the idea of electrophilicity and nucleophilicity of the synthesized compounds, and it was envisaged that depression of electron density is highly dependent on the substituent present on an aromatic ring. The first hyperpolarizability values showed that all synthesized derivatives has a potential non-linear optics (NLO) response, but **5c** has a significant NLO potential due to its large βo (1373.76 au) value.

## Figures and Tables

**Table 1:** Suzuki–Miyaura reaction of 2-bromo-4-chlorophenyl-2-bromobutyrate with different phenyl boronic acids.

Sr. No.	Phenyl Boronic Acid	Products	Solvent/H_2_O (4:1)	Yield %
**5a**	3-Cl-4-F-C_6_H_4_	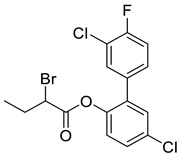	1,4-dioxane	64
**5b**	3-(MeCO_2_)-C_6_H_4_	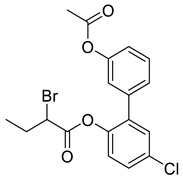	1,4-dioxane	76
**5c**	4-(MeS)-C_6_H_4_	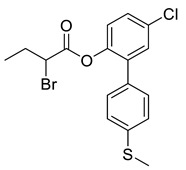	1,4-dioxane	79
**5d**	3,4-Cl_2_-C_6_H_4_	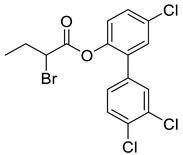	1,4-dioxane	68
**5e**	4-MeO-C_6_H_4_	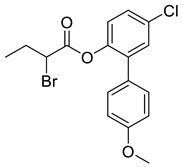	1,4-dioxane	81
**5f**	3-Cl-C_6_H_4_	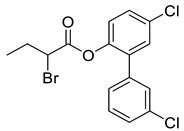	1,4-dioxane	73

**Table 2:** The energies of HOMOs (E_HOMOs_, in eV), energies of LUMOs (E_LUMOs_, in eV,) HOMOs–LUMOs gap (G_H-L_), and dipole moment (μ, in Debye) of compounds (**5a**–**f**).

Compounds	E_HOMOs_	E_LUMOs_	G_H-L_
**5a**	−7.06	−1.93	5.14
**5b**	−7.03	−1.97	5.06
**5c**	−6.06	−1.79	4.27
**5d**	−7.07	−1.98	5.09
**5e**	−6.30	−1.73	4.57
**5f**	−7.06	−1.87	5.19

**Table 3:** Ionization potential (*I*), electron affinity (*E_A_*), chemical hardness (Ƞ), electronic chemical potential (μ), electrophilicity index (Ꙍ), and nucleophilicity (N) of compounds (**5a**–**f**). All values are given in eV.

Compounds	Ionization Potential (*I*)	Electron Affinity (*E_A_*)	Chemical Hardness (Ƞ)	Electronic Chemical Potential (μ)	Electrophilicity Index (Ꙍ)	Nucleophilicity (N)
**5a**	7.06	1.93	2.57	−4.50	3.94	5.14
**5b**	7.03	1.97	2.53	−4.50	4.00	5.06
**5c**	6.06	1.79	2.13	−3.92	3.61	4.27
**5d**	7.07	1.98	2.55	−4.52	4.02	5.09
**5e**	6.30	1.73	2.29	−4.01	3.52	4.57
**5f**	7.06	1.87	2.60	−4.46	3.83	5.19

**Table 4:** Molecular electrostatic potential (MEP) values of all compounds (**5a**–**f**).

Compounds	−ve Potential	+ve Potential
**5a**	−4.77 × 10^−2^	4.77 × 10^−2^
**5b**	−5.01 × 10^−2^	5.01 × 10^−2^
**5c**	−5.13 × 10^−2^	5.13 × 10^−2^
**5d**	−4.75 × 10^−2^	4.75 × 10^−2^
**5e**	−5.22 × 10^−2^	5.22 × 10^−2^
**5f**	−4.95 × 10^−2^	4.95 × 10^−2^

**Table 5:** Polarizability (α_o_) and hyperpolarizability (β_o_) of compounds **5a**–**f** with CAM-B3LYP and LC-BLYP methods.

Compounds	CAM-B3LYP		LC-BLYP	
	α_o_	β_o_	α_o_	β_o_
**5a**	236	515.43	229	436.87
**5b**	252	284.34	244	223.17
**5c**	264	1373.76	255	1010.54
**5d**	250	539.03	243	445.12
**5e**	244	954.47	237	776.21
**5f**	235	218.51	228	173.78

**Table 6:** The static, dc-Kerr effect, electric field-induced second harmonic generation coefficients (in au), and quadratic non-linear refractive index (*n*_2_ = cm^2^/W) at different wavelengths (in nm) for compounds (**5a**–**f**).

Compounds	Frequency	*γ*(0; 0, 0, 0)	*γ*(−ω; ω, ω, 0)	*γ*(−2ω; ω, ω, 0)	*n*_2_
**5a**	0	48741.5			
	532		64320.8	199529	8.63 × 10^−18^
	1064		51988.4	59645.3	4.43 × 10^−18^
**5b**	0	54142.2			
	532		73258.1	293432	1.16 × 10^−17^
	1064		58215.3	67505.4	4.96 × 10^−18^
**5c**	0	86291.9			
	532		141361	34064301	9.46 × 10^−16^
	1064		95936.8	122589	8.41 × 10^−18^
**5d**	0	57794.5			
	532		78682.2	314130	1.24 × 10^−17^
	1064		61860	72208.8	5.3 × 10^−18^
**5e**	0	63232			
	532		92924.8	1013010.1	3.23 × 10^−17^
	1064		69085.6	83451.7	5.96 × 10^−18^
**5f**	0	49285.2			
	532		64440.9	176408	8.01 × 10^−18^
	1064		52669.5	60014.7	4.47 × 10^−18^
